# Specification of Change Mechanisms in Pregnant Smokers for Malleable Target Identification: A Novel Approach to a Tenacious Public Health Problem

**DOI:** 10.3389/fpubh.2017.00239

**Published:** 2017-09-19

**Authors:** Suena H. Massey, Jean Decety, Katherine L. Wisner, Lauren S. Wakschlag

**Affiliations:** ^1^Department of Psychiatry and Behavioral Sciences, Northwestern University Feinberg School of Medicine, Chicago, IL, United States; ^2^Institute for Innovations in Developmental Sciences, Northwestern University, Evanston, IL, United States; ^3^Department of Psychology, University of Chicago, Chicago, IL, United States; ^4^Department of Medical Social Sciences, Northwestern University Feinberg School of Medicine, Chicago, IL, United States

**Keywords:** pregnancy, smoking cessation interventions, addiction, empathy, attachment, oxytocin, *OXTR*, behavior change

## Abstract

Maternal smoking during pregnancy (MSDP) continues to be a leading modifiable risk factor for perinatal complications and a range of neurodevelopmental and cardio-metabolic outcomes across the lifespan. Despite 40 years of intervention research less than one in five pregnant smokers who receive an intervention quit by delivery. Within this context, recognition of pregnancy is commonly associated with abrupt suspension or reduction of smoking in the *absence* of intervention, yet has not been investigated as a volitional target. The goal of this article is to provide the empirical foundation for a novel direction of research aimed at identifying malleable targets for intervention through the specification of behavior change mechanisms specific to pregnant women. To do so, we: (1) summarize progress on MSDP in the United States generated from conventional empirical approaches to health behavior change; (2) discuss the phenomenon of spontaneous change in the absence of intervention among pregnant smokers to illustrate the need for mechanistic specification of behavior change motivated by concern for fetal well-being; (3) summarize component processes in neurobiological models of parental and non-parental social behaviors as a conceptual framework for understanding change mechanisms during pregnancy; (4) discuss the evidence for the malleability of these processes to support their translational relevance for preventive interventions; and (5) propose a roadmap for validating the proposed change mechanism using an experimental medicine approach. A greater understanding of social and interpersonal processes that facilitate health behavior change among expectant mothers and how these processes differ interindividually could yield novel volitional targets for prenatal interventions. More broadly, explicating other-oriented mechanisms of behavior change during pregnancy could serve as a paradigm for understanding how social and interpersonal processes positively influence health behaviors across the lifespan.

## Health Behaviors are Difficult to Change

Despite substantial research, maternal smoking during pregnancy (MSDP) continues to be a leading modifiable contributor to low-birth weight, a robust risk factor for adverse neonatal, neurodevelopmental, metabolic, and cardiovascular outcomes across the lifespan (1). The year 2017 marks the 40th anniversary since publication of the first prenatal smoking cessation intervention trial (2). Since this time, 96 randomized controlled intervention trials (RCTs) including over 31,000 pregnant smokers, testing 37 individual behavior change techniques (3, 4), and an additional 26 qualitative studies (5, 6) have led to important but modest gains. In the United States, the prevalence of MSDP fell most dramatically in the 1990s from 17.3 to 13.3%. However, in the following decade, the prevalence fell by just 1 to 12.3% in 2010 (7). This means that one in nine infants in the United States is born prenatally exposed to MSDP. This figure is as high as one in four for infants born to socioeconomically disadvantaged women and women from marginalized populations (7, 8). On average, intervention increases the chances a pregnant smoker will stop smoking before delivery by only 6% (4). Even for interventions involving financial incentives for biologically verified abstinence, well under half of pregnant smokers achieve abstinence by delivery (9). Intervention—especially financial-incentive-based interventions—*are* associated with improved birth outcomes (10). However, to achieve the goal of 1% prevalence in MSDP set by Healthy People 2020, an infusion of novel approaches is imperative (11).

With this goal in mind, we reflect on MSDP within the broader challenge of promoting healthy lifestyles in the general population that spans at least a half a century (12). Following the development of 83 largely overlapping theories of behavior change comprising more than 1,000 constructs and 23 different intervention targets (13), the leading causes of morbidity and mortality in the United States continue to be directly attributable to lifestyle behavior, namely, smoking, poor diet, and inactivity (14, 15). For example, when we consider that obesity has become more rather than less prevalent during this time, and is presenting earlier in life in the United States (16, 17), the progress made on reducing MSDP seems outstanding in comparison. And this is hardly surprising.

*Pregnancy itself elicits abrupt temporary change in smoking behavior without formal intervention and does so more effectively than any intervention strategy tested to date* (18). The presence of an other-oriented mechanism of change—concern about the unborn child—is well-documented in qualitative (5) and quantitative studies (3, 19, 20), yet remains poorly understood. This unique interpersonal aspect of MSDP could represent a powerful volitional target for intervention.

We describe a novel conceptual framework for mechanistic research on health behavior change motivated by concern for others, as is observed in pregnant smokers. Foundational to the proposed framework are cognitive, affective, motivational, and regulatory processes described in emerging neurobiological models of human caregiving behavior and how these processes interact dynamically with addictive processes. We illustrate the potential utility of this proposed mechanism, if validated, for intervention development and personalization of treatment. We conclude by outlining a roadmap for empirical validation of the proposed mechanism and its application to intervention development. A systematic review of the literature is not the aim of this article—we refer readers to recent high-quality systematic reviews of prenatal smoking cessation intervention studies (3); qualitative studies on MSDP (5, 6); and other relevant topics throughout this article.

## Why Study Change Mechanisms?

Traditionally, health behavior change research has involved efficacy trials aimed at modifying one or more targets identified from behavior change theories [for a full taxonomy of these theories see Ref (13).]. In recent years, recognition of the limitations and inefficiency of this approach has fueled interest in an *Experimental Medicine* (EM) approach that prioritizes a mechanistic understanding of putative targets prior to conducting full efficacy trials (21). Clearly elucidated mechanisms of change enable scientists to conclude why a particular intervention worked or failed to work in a given study and affords the opportunity to translate knowledge gained from one particular trial to other contexts, behaviors, or populations (13). Moreover, behavior change theories are limited by (a) a lack of specification of population and context (i.e., in what populations or contexts are theories and targets identified by theories most predictive of behavior change); (b) a lack of are specification of volitional factors (why people would want to change in the first place); (c) the inability to infer how change in targets over time influences behavior since theories are derived primarily from correlational evidence; and (d) the rare examination of affective processes as putative targets for interventions (22).

Lack of specification is reflected in the U.S. Preventive Services Task Force’s (USPSTF) recommendation for “*Tobacco Smoking Cessation in Adults, Including Pregnant Women*” using the 5 A’s intervention (Ask, Assess, Advise, Assist, and Arrange). The extent of specification for pregnant women involves (a) behavioral rather than pharmacologic interventions and (b) ensuring educational materials about risk are pregnancy-specific (23, 24). There are clearly overlaps in barriers to smoking cessation among non-pregnant and pregnant women. These include stress and its psychopathological manifestations (25, 26), limited internal and external resources (27, 28), and smoking-related factors (29, 30). However, smoking cessation among pregnant women is also associated with unique volitional factors. It is externally- rather than internally-motivated and appears to be temporary (19, 31). Yet, these unique factors have not been adequately explicated as change mechanisms.

## Other-Oriented Concern as a Putative Target for Behavior Change Interventions

The term “spontaneous quitters” has been used to describe a substantial subset of pregnant smokers—some 40–65% of privately insured smokers and 11–28% of smokers in publicly funded obstetric clinics—who stop smoking without intervention in early pregnancy and prior to the onset of obstetric care in the United States. The prevalence of spontaneous quitting is remarkably similar across the United Kingdom and other European countries (32, 33). Spontaneous quitters have lower smoking-related risks (i.e., nicotine dependence, partner smoking, age of smoking initiation, impulsivity) and comparatively favorable demographic profiles (being married, more highly educated, and nulliparous) (34). However, there is converging evidence that unmeasured factors or processes beyond the mere absence of risks facilitate sustained abstinence among spontaneous quitters.

First, spontaneous quitters achieve abstinence rates two to three times higher than non-pregnant women who quit “cold turkey” over a similar period of time (65–81% across gestation when biochemically verified) (34–36). Spontaneous quitters’ abstinence rates also compare favorably to other highly motivated populations including men and women who recently suffered a heart attack (37, 38). Moreover, spontaneous quitters report greater confidence about their ability to remain abstinent for the duration of pregnancy relative to non-pregnant quitters over the same period of time, yet do not use the behavioral strategies (i.e., coping skills, distractions, oral-substitutes) used by successful recent ex-smokers (39).

Even women who make adoption placements at birth report suspending tobacco, alcohol, and illicit drug use at rates comparable to rates reported in epidemiologic samples (28, 40, 41). This suggests that mechanisms that facilitate spontaneous quitters’ success are at least partially attributable to *being pregnant*, distinct from factors associated with the transition to parenthood (42). Moreover, these mechanisms may apply to other addictive behaviors besides cigarette smoking (43). Consistent with this are findings by Curry and colleagues (31) that pregnant smokers endorse different motives for abstinence from smoking during pregnancy, i.e., “*I want to be healthy for my baby,”* versus abstinence during the postpartum period “*I do not want to be known as a smoker by my child”* (31, 44).

Finally, the mechanisms that facilitate spontaneous quitting could reflect a broader pattern of harm reduction. Substantial inter- and *intra-*individual fluctuation in smoking across gestation has been documented in quantitative studies of pregnant women (45). In qualitative studies, many women who do not abstain from smoking during pregnancy reduce their smoking early in pregnancy with the stated intent of reducing harm to the fetus (since quitting altogether is not perceived as possible) (3, 46, 47). Additionally, the power of the imagined harm to the fetus caused by continued smoking during gestation is evidenced by intense guilt reported by women who cut down but do not abstain (5). In one of the largest studies specifically focused on spontaneous quitting which included 118 spontaneous quitters and 231 continuing smokers, the belief that “smoking will greatly harm my baby” was associated with a 14-fold increase in the likelihood of being a spontaneous quitter (27). In summary, in the absence of intervention, “being pregnant” seems to positively influence smoking trajectories *via* temporary suspension and reduction of smoking. Elucidating this change mechanism and how it can be amplified can yield new avenues for improving the immediate and long-term health of mothers and their children.

To do so, we take an interdisciplinary approach that blends advances in social neuroscience (48), parenting neurobiology (49), and the interruption of parenting processes by addiction (50). Drawing from these literatures, we hypothesize that key cognitive, affective, motivational, and regulatory processes that facilitate responsive caregiving behavior, as characterized across separate theoretical foundations (51–53), may be highly relevant for identifying putative targets for intervention during pregnancy. Moreover, we illustrate how this developmental framework, if validated, can be used to personalize interventions. Finally, we describe a detailed research agenda aimed at validating the proposed mechanism.

## Models of Caregiving Behavior

Striking similarities in the mechanisms of affiliation and maternal caregiving behavior across mammalian species and their modulation by the neuropeptide hormone oxytocin support their highly conserved role in survival, adaptation, and evolution (54). In recent decades, functional magnetic neuroimaging (fMRI) studies in human parents presented with simulated cues salient to caregiving (i.e., infant faces, cries, and odors) have facilitated the identification of a network of neural regions implicated in parental caregiving commonly referred to as a *parenting network, parenting circuitry*, or the *“parental brain”* [for a recent review of these fMRI studies, see Ref (55)]. Optimal spatial resolution offered by fMRI is nicely complemented by temporally sensitive neuroimaging techniques such as electroencephalography (EEG) with event-related potentials (ERPs) and magneto-encephalography from which processing functions of specific neural structures may be inferred. For example, the temporal sequence of signals following an experimental stimulus is used to infer sensory perception, detection of salience, and attentional processes, followed by “top-down” regulation and reappraisal (56). Neural processes associated with parenting behavior, combined with their regulation by oxytocinergic, hypothalamic–pituitary–adrenal axis and autonomic functions are referred to, collectively, as the *caregiving system* (49, 51). This system is also thought to regulate altruistic (other-oriented) prosocial (helping) behaviors outside of the parenting context (51, 57–59).

Common to both sensitive parenting behavior and prosocial behavior is *empathy* (60), defined here, as the ability to perceive others’ need, coupled with a motivation to help (61, 62). Different definitions of empathy within and across scientific disciplines have created confusion, detracting from its highly conserved and critical role in social behavior (48, 58, 63). The dual function of empathy-related mechanisms in promoting other-oriented responding while regulating internal stress responses is, however, increasingly recognized (though not explicitly) by intense inquiry regarding the therapeutic potential of its biological substrate, oxytocin (48, 49, 64). For heuristic purposes, we will use the terms, *caregiving processes* and *empathic processes* interchangeably to refer collectively, to the perceptual, affective, motivational, and regulatory processes necessary for the expression of behavior aimed at helping others in parental and non-parental contexts, respectively. Specific cognitive and affective facets of empathy (i.e., affective sharing, empathic concern, perspective taking or cognitive empathy, and personal distress) will also be defined herein to illustrate how they map onto perceptual, affective, motivational, and regulatory processes described in parental caregiving models.

## Specific Processes that Facilitate Caregiving

The component processes involved in parental caregiving behavior may be broadly characterized as: (a) the perception of cues signaling need or distress in another; (b) affective processing of these cues; (c) competing other-oriented versus self-oriented motivational states; and (d) subsequent behaviors (to help versus to withdraw) moderated by internal regulatory capacity (51, 65, 66). Below, we elaborate on these processes and describe how they may facilitate motivation to reduce or suspend smoking following the recognition of pregnancy.

### Perception of Others’ Need or Distress

The ability to detect distress in others (and the subsequent motivation to help) is observable in young children including infants prior to the development of more complex social cognitive abilities (62, 67, 68). Qualitative studies suggest that the recognition of a pregnancy is a highly salient event (69). As fetal needs are perceived in abstract terms, rather than seen or heard as is the case with infant cues following delivery, the capacity and tendency to imagine others’ perspective, described by the construct *perspective taking* (58), could influence the degree to which caretaking processes are activated in pregnant smokers. In fact, there is preliminary evidence for the role of perception of others’ distress in MSDP. Among women who possess *OXTR* variants previously associated with increased sensitivity to social cues (70, 71), the ability to accurately identify distress in others has been associated with lower levels of biochemically assessed smoking across pregnancy (72). Following the perception of need in others, subsequent affective processing of this information critically predicts divergent behavioral responses. This is described in models of social behavior derived from research on parenting neurobiology, social neuroscience, and human attachment and affiliation described below.

### Affective Processing of Cues Signaling Others’ Need or Distress

#### Affective Processing in the Parental Caregiving System

Rodent models of caregiving support the roles of specific nuclei in the hypothalamus and oxytocin in caretaking through (a) enhancing *approach motivation* (to help offspring in need) involving the nucleus accumbens-ventral pallidum reward circuit; while (b) inhibiting a competing *avoidance motivation* (to withdraw from offspring) by inhibiting the transmission of threat signals from the amygdala to the periaqueductal gray (73). A similar dynamic of competing motivations and their modulation by oxytocin has since been supported by neuroimaging studies in humans (74, 75). In a particular RCT, intranasal oxytocin administration in mothers presented with infant stimuli was associated with increased insular activation (supporting caregiving processes), concomitant with reduced activation of the amygdala (involved in the processing of threat) (76). This competing dynamic of other-oriented versus self-oriented processes may be highly relevant for understanding the ambivalence verbalized by pregnant smokers, who describe the competing desires to reduce harm to the fetus, yet still maintain their own emotional stability by continuing to smoke (5, 6). The child development literature similarly acknowledges how parents’ emotional control is tightly intertwined with cognitive control (i.e., executive function) that together, predict sensitivity of parenting behavior [for a review, see Ref (66)].

#### Affective Processing in Social Neuroscience Models

In affective neuroscience models, affective processing involves two functionally distinct processes—*affective sharing* and *empathic concern*—quantified using behavioral paradigms involving images of others’ limbs in painful contexts (hand under a knife or a bare foot under a car tire) (53, 57). Affective sharing describes a reflexive somatovisceral resonance with others’ pain while maintaining self-other distinction, corresponding to an early N200 signal on EEG/ERPs occurring within 160–220 ms following a stimulus. Affective sharing of others’ pain results in activation of neural circuitry involved in one’s own experience of pain as detected using fMRI, i.e., the anterior insula, anterior cingulate cortex, supplementary motor area, amygdala, somatosensory cortex, and periaqueductal gray (61, 77).

Broadly consistent with the concept of competing approach and avoidance motivations described in parental caregiving models (51), whether affective sharing leads to other-oriented empathic concern and motivation to help, versus self-oriented *personal distress* (58) and motivation to withdraw, is also thought to be dependent on top-down regulatory processes. Specifically, empathic concern is associated with a late positive potential on EEG/ERP, occurring 400–800 ms following stimulus presentation indicative of cognitive reappraisal and emotional regulatory processes (78, 79). There is also evidence that the capacity for perspective taking (imagining others’ point of view) is associated with lower stress reactivity, whereas personal distress is associated with higher stress reactivity, respectively (80). This parallels mechanisms of stress attenuation that facilitate approach motivation described in the in caregiving system, and supports a dynamic interplay between stress and caregiving processes (81).

#### Affective Processing in Attachment Research

The closeness, or affiliation felt toward another, described by *attachment*, is another facet of affective processing hypothesized to influence smoking behavior during pregnancy. The neurobiological understanding of human parental attachment is derived from functional neuroimaging paradigms comparing neural responses to parents’ own infant’s stimuli to responses to other infants’ stimuli (82, 83). There is broad recognition that attachment develops during gestation, termed *maternal fetal attachment* (84). Greater maternal fetal attachment in pregnant women has been linked to increasing plasma oxytocin levels across gestation (85), more positive health-related behaviors during pregnancy [see Ref (86) for a review], including smoking cessation during pregnancy (20) and consumption of fewer cigarettes per day among persistent pregnancy smokers (87).

Maternal–child attachment is thought to emerge from mothers’ thoughts and fantasies about their children, called *representations* (88), beginning during pregnancy (i.e., prenatal representations*)* (89), assessed by structured interviews (90). Prenatal representations could provide an avenue for improving maternal fetal attachment, and putatively, health-related behaviors. In attachment-theory-based research, cognitive empathic abilities are described by *reflective function* and *mind-mindedness*. Reflective function describes the capacity to accurately interpret others’ non-verbal cues enabling sensitive and contingent responding to others’ needs (91). Intact reflective function requires mind-mindedness or the ability to interpret others’ behaviors in the context of their thoughts and emotions. Mothers who possess mind-mindedness are able to imagine thoughts and emotions in their children that govern their behaviors (92). Within this theoretical framework, underlying mechanisms of unresponsive or deficient parenting (i.e., neglect, use of harsh disciplinary practices) among mothers with substance use disorders are beginning to be elucidated.

## How Addictive Processes may Interact with Caregiving Processes

### Impaired Caregiving in Mothers with Substance Use Disorders

Women who smoked persistently during pregnancy (in the absence of illicit substance abuse) are also more likely to exhibit harsh parenting practices though the underlying mechanisms are not well elucidated (93, 94). Research on mothers with cocaine and opiate use disorders, however, provides possible explanations (95). Studies have shown that impaired reflective function in these mothers (91, 96) underlies maltreatment and neglect (97). Differences in the regulation of reward and stress have also been posited as explanations in light of the substantial overlap in neural circuitry implicated in parenting behavior, reward processing, and regulation of stress (98). Substance-using mothers exhibit reduced activation in parenting circuitry when presented with infant cues suggestive of an attenuated empathic response (99, 100). These findings are broadly consistent with neurobiological models of addiction in which socially salient cues fail to signal reward due to drug-induced allostatic modifications related to prolonged drug exposure (101, 102). In this way, parenting cues that might normally activate reward pathways in non-using mothers may alternatively trigger stress responses in addicted mothers and increase vulnerability to relapse as a form of coping (98).

### Structural and Functional Alterations in Empathic Processing in Non-Pregnant Smokers and Substance Users

There is converging evidence for impaired social information processing in non-pregnant smokers and substance abusers though whether impairments are a cause or consequence of smoking and substance use (or some combination) is unknown [for a reviews see Ref (103, 104)]. Chronic heavy smokers show reduced gray matter volume in empathic processing regions relative to individuals who never smoked (103). Resting-state functional connectivity in the anterior insula-ventromedial prefrontal cortical circuit also differs among non-pregnant individuals with and without nicotine dependence (105). Weakened connectivity in this circuit fully mediated a found link between alexithymia (inability to describe one’s own emotions) and cigarette craving in one study of 24 smokers and 20 non-smokers (106). Deficits in affective and cognitive empathy have also been documented in a number of cross-sectional studies of non-pregnant women and men with alcohol, cocaine, and methamphetamine use disorders, with unclear directionality in the addiction-empathy link (104).

If pregnant smokers have impairments in empathy as might be suggested by these studies, according to the hypothesized caregiving mechanism, cues salient to caregiving such as the recognition of pregnancy or advice from providers to quit, may not be processed in the same way as in women who never smoked. This could also explain the apparent resilience to intervention observed among pregnant smokers who smoke heavily (107). Namely, the motivation to protect the fetus—assumed to be present in all pregnant smokers, *may actually be attenuated due to neuroadaptations related to chronic smoking itself*, rendering heavy smokers no more likely to quit than non-pregnant individuals (103, 105). We go one step further to illustrate how a caregiving mechanism could explain individual differences in responses to interventions.

### How Processing Differences Might Explain Varied Responses to MSDP Interventions

An interaction between nicotine dependence and motivation to quit smoking was suggested by Stotts and colleagues in their 2009 study that tested an innovative 30-min therapeutic fetal ultrasound session (U/S). The U/S session was aimed at providing a biological marker of risk hypothesized to increase motivation for smoking cessation (108). Arms of this intervention trial were: (a) U/S with motivational interviewing (U/S + MI); (b) U/S with the best practices 5 A’s intervention (U/S + BP); and (c) the 5 A’s alone (BP) (109). Differences in cessation rates by intervention group were not found for the entire sample of *N* = 360 pregnant smokers.

However, *post hoc* analyses showed that women who smoked less than 10 cigarettes/day (light smokers) appeared to benefit from the U/S intervention; cessation rates for U/S + MI, U/S + BP, and BP alone were 34, 25.8, and 15.6%, respectively. For women smoking 10 or more cigarettes/day (heavy smokers), cessation rates were 0% for U/S + MI, 2.3% for U/S + BP, and 7% for BP alone. Thus, heavy smokers who received U/S appeared to be *less* likely to quit smoking relative to heavy smokers receiving the less intensive 5 A’s intervention. Authors hypothesized that the U/S could have had an opposite effect as intended—of reassuring smokers about the well-being of the fetus.

Interpreting these results using a caregiving framework, we posit that for heavy smokers, visualization of the fetus during the U/S (a visual cue salient to caregiving) failed to elicit its intended effect on smoking behavior due to attenuated empathic responding, much as neural responses to infant stimuli are attenuated among smoking and substance-abusing mothers (99, 100). On the other hand, applying the model of competing approach versus avoidance motivation described in the caregiving model (51), it is also conceivable that the U/S intervention had the effect of enhancing self-oriented personal distress and guilt associated with continued smoking in heavy smokers rather than promoting other-oriented motivation to quit (6) as intended. This could have led to continued smoking to cope with guilt. Fortunately, there is growing support for the malleability of empathic processes *via* behavioral interventions.

## Evidence for the Malleability of Empathic Processes

### Attachment-Based Interventions to Increase Reflective Function

Parenting interventions designed to target impaired reflective function [i.e., *Minding the Baby* (110)] are effective for improving the sensitivity of subsequent parenting behavior, the quality of mother–child interactions, reward experienced by mothers when interacting with their children, and also the chances of maintaining abstinence from drug use (111, 112). Results from these programs support not only the malleability of empathic processes, then, but also the possibility that attachment processes effectively compete with addictive processes.

### Mindfulness-Based Interventions That Increase Empathy

There is intense interest in mindfulness-based interventions for smoking cessation and substance misuse (113, 114). One particular mindfulness-based exercise aimed at promoting caring and compassion toward oneself and others called loving kindness mediation (LKM) may hold promise for MSDP if hypothesized change mechanisms are validated. Based on recent reviews of this young literature, LMK appears to improve perceived well-being, biological markers of stress, and behavioral control in non-pregnant individuals through increases in positive emotions and empathy (115–117). The impact of LKM on a range of outcomes seems robust with minimal intervention. A recent study showed that simply listening to LKM language once, *without actually mediating*, increased both reported empathy and ratings of sensitivity to others’ pain when compared to a control condition that involved exposure to similar language but replaced words connoting *love* with words connoting physical safety and security (118).

### Experimental Pharmacologic Interventions in Non-Pregnant Individuals

The documented effects of pharmacologic interventions aimed at increasing empathy, while not necessarily appropriate for pregnant women, provide further support for the malleability of caregiving and empathic processes. In a growing number of studies of non-pregnant healthy and disease populations, one-time administration of intranasal oxytocin seems to increase empathic ability, prosocial behaviors, and to reduce stress reactivity. The underlying mechanisms and safety of intranasal oxytocin has yet to be established, however, even for healthy non-pregnant individuals [Ref. (119), for a review]. Nonetheless, there is also increasing interest in how therapeutic interventions involving oxytocin could be used in the treatment of addiction. Intranasal oxytocin administration in non-pregnant smokers has been shown to reduce cue-elicited craving (120) suggesting that the interaction between affiliative processes and addictive processes may be bidirectional (104, 121). Intranasal oxytocin also reduces craving and stress responses in marijuana-dependent individuals (122), reduces drug-seeking among cocaine dependent individuals (123), and reduces withdrawal symptoms and benzodiazepine requirements among alcohol dependent individuals (124). Similar effects have also been observed with exogenous administration of 3,4-methylenedioxymethamphetamine (MDMA), commonly known as the recreational drug, ecstasy (125). Finally, polymorphisms in the oxytocin receptor gene (*OXTR*) previously linked to sensitivity to social context (126–128), and implicated in MSDP (72), appear to predict which individuals will respond most robustly to all three of the aforementioned empathy-enhancing modalities (LKM, oxytocin, and MDMA), raising the possibility of a personalized approach to MSDP in the future.

## The Expectant Brain as the Next Horizon in Prevention

Whether and to what extent the caregiving processes described in parents occur during pregnancy is not clear due to the relative paucity of clinical research studies conducted in pregnant women (129). However, tremendous plasticity observed during the early postpartum period (130) and the prediction of maternal–infant attachment by maternal–fetal attachment and plasma oxytocin during pregnancy support the emergence of caregiving processes prenatally (85). There is also early evidence that pregnant women show heightened vigilance to novelty and threat cues close to delivery when compared with non-pregnant women (131–133).

The most compelling evidence for prenatal neuroadaptions to caregiving comes from a recent longitudinal structural MRI study. Comparison of pre-pregnancy and postpartum scans showed reductions in gray matter volume in regions implicated in social information processing (i.e., the theory of mind network) among primiparous pregnant women. These structural changes predicted observed and reported attachment to infants postpartum and were neither observed in women’s male partners, multiparous pregnant women, nor in a comparison sample of non-pregnant women over the same period of time (134). These findings suggest that structural changes related to postpartum caregiving occur during a woman’s first pregnancy but not in successive pregnancies. *Could this observed neuroplasticity during a woman’s first pregnancy facilitate spontaneous behavior change?*

Increasingly sophisticated methods for assessing the maternal–fetal dynamic *via* fetal neurobehavioral assessments have led to a greater understanding of the complex mechanisms through which maternal mood and behavior during pregnancy influence fetal neurodevelopment and long-term developmental outcomes for children (135, 136). These approaches can also provide rich experimental and therapeutic opportunities for intervention on MSDP. Realizing the potential scientific gains from these diverse empirical frameworks, however, requires scientific collaboration that transcends disciplines and methodologic approaches (137). These disciplines include but are not limited to the science of behavior change, parenting and addiction neurobiology, attachment theory, social neuroscience, developmental psychology, and developmental neurobiology. With this spirit of interdisciplinary collaboration in mind, we present a research agenda for the application of this collective knowledge toward identifying novel targets for prenatal smoking cessation interventions.

## A Roadmap for Research Using an EM Approach

The Science of Behavior Change working group of the National Institutes of Health recommends the following 4-step EM approach that focuses on the importance of understanding behavior change mechanisms prior to conducting efficacy trials (21).

(1) Identify a modifiable factor that relates to behavior as a putative target for intervention.
(2) Validate the putative target through the development of a multilevel assay of the target to assess when, how, and to what extent the target elicits change in the behavior.
(3) Determine the intervention(s) that best engage(s) and produces the desired change in the target.
(4) Conduct a RCT to determine whether the intervention changes the behavior *via* its effect on the target.

### Step 1: Empathic Processes as Putative Target(s) for Intervention

The qualitative and quantitative research on MSDP (3, 5, 6, 27) and individual studies linking maternal fetal attachment and empathic processes with smoking cessation among pregnant women described in Sections “Other-Oriented Concern As a Putative Target for Behavior Change Interventions,” “Models of Caregiving Behavior,” “Specific Processes That Facilitate Caregiving,” “How Addictive Processes May Interact with Caregiving Processes,” and “Evidence for the Malleability of Empathic Processes,” above (20, 72, 87) provide the empirical foundation for empathic processes as putative targets for intervention.

### Step 2: Development of a Multilevel Empathy Assessment Battery

To ensure valid measurement of target engagement in intervention trials, incorporation of reported, behavioral, neuroimaging, and biological measures is ideal (21). Measures that most robustly and reliably either: (a) differentiate spontaneous quitters and persistent smokers and/or (b) predict the degree of change in smoking should be selected for use in a multilevel assay.

#### Behavioral Measures

Empathic processes assessed using behavioral paradigms of affective empathy (images of others’ pain), other measures of social cognition such as face emotion processing and parental attachment paradigms (infant cues) should be examined in relation to prospectively assessed biochemically verified patterns of smoking behavior across pregnancy. Incorporation of neuroimaging during the administration of performance-based measures would be ideal as it could verify target engagement at the neuroanatomical level. Different imaging approaches offer different benefits and liabilities. While fMRI would provide optimal spatial resolution, it is more costly, less convenient, and may be less acceptable to pregnant women (138). EEG/ERP is more feasible and provides optimal temporal resolution, but poor spatial resolution. Finally, directly observed measures of empathic behavior such as a social discounting task previously linked to MSDP (139) and postpartum observations of mother–infant interactions (140) may also be useful.

#### Biological Measures

Non-invasive physiologic tests can detect the magnitude of general affective responding to stimuli presented in behavioral paradigms. Vagal (parasympathetic) tone is a non-specific measure of a range of health-related outcomes including compassion for the suffering of others (141), ascertained through heart rate variability from electrocardiographic monitoring. Electrodermal monitoring detects changes in skin conductance associated with acute sympathetic responses to stress delivered by experimental stimuli. Measurement of peripheral oxytocin and cortisol levels at baseline and before and after an experimental stimulus could be useful as an additional biological measure of empathic and stress responding. However, a number of methodologic challenges related to pulsatile release and diurnal variation need to be carefully considered in study design and interpretation of results (142, 143).

#### Other Methodological Considerations

The multilevel assay for empathic processes should be conducted in a sample of pregnant smokers who quit following recognition of pregnancy and those who did not, ideally matched on demographic and other characteristics associated with MSDP, and beginning as early in pregnancy as possible. Assessment of empathy prior to conception, for example, in a population of sexually active smokers of child-bearing age would be ideal, but challenging. Conducting the assay for empathy-related processes three or more times across gestation would enable the assessment of change across pregnancy. Measures that most robustly differentiate quitters from persistent smokers or relate to the degree of change (for example, reduction in cigarettes per day following recognition of pregnancy) should be selected for use in tests of target engagement, evaluation of interventions, and eventually, full efficacy trials.

We stress the importance of examining continuous measures of smoking and more nuanced smoking patterns beyond standard categorizations (i.e., number of quit attempts among those not achieving sustained abstinence and the degree of reduction or increase) to not only capture fluctuation in smoking (45) but also to quantify efforts made to change behavior. Timeline follow back methods (interview or computer-assisted) to capture daily smoking (144) combined with periodic cotinine verification adjusted for trimester of pregnancy (145) and reporting error (146) remains the gold standard for prenatal smoking assessments.

Ecological momentary assessment (EMA) is a popular tool that provides extremely fine-grained information about constructs of interest in relation to behavior (147). However, EMA activities could serve as a cue that influences smoking behavior similar to the way that behavior monitoring approaches (i.e., food diaries) can change behavior over time (148). This could limit the validity of data collected for observational purposes. Wireless physiologic monitoring devices now widely available (149), however, might be a feasible approach to monitoring biomarkers such as vagal tone and stress reactivity in conjunction with periodic reports since these devices can record data continuously with minimal action required by the wearer.

## Conclusion: A Call to Action on Prenatal Preventive Intervention

A mother’s behavior during pregnancy is arguably the most robust modifiable determinant of the intrauterine environment, increasingly recognized as a critical contributor to the lifelong health of her unborn child (150, 151). In this way, improving the impact of prenatal intervention constitutes a far-reaching preventive investment (152). The challenges of behavior change, even during the uniquely opportune window of pregnancy, however, are substantial and felt by pregnant women, their health-care providers, and intervention scientists alike. Pregnant women who smoke and use other addictive substances face enormous biopsychosocial risks that are often perpetuated inter-generationally. These risks compound the effects of smoking on mothers’ own health and the health of their families. A deeper mechanistic understanding of MSDP that transcends interdisciplinary divisions to leverage scientific advances generated from multiple perspectives and theoretical foundations is proposed to most effectively and efficiently address this major public health problem.

We underscore the importance of scientific progress on MSDP made in the past 40 years. Intervention is associated with increases in birth weight even when total abstinence is not achieved. Moreover, interventions that provide financial incentives for confirmed abstinence (the most effective approach to date) are clearly cost-saving when considering the neonatal health-care costs saved (3, 10). But this may not be a satisfactory solution. The long-term ethical, policy, and personal implications of widespread financial incentive-based interventions for MSDP are unknown. Perceptions regarding the use of public resources for these interventions could compound the stigma and marginalization already felt by populations disproportionately affected by MSDP leading to more harm than benefit (6). Moreover, reducing prenatal smoking exposure alone may not reduce children’s long-term mental health risks. Unlike animal models, in which prenatal nicotine exposure has demonstrated clear and causal effects on offspring brain development, causality in humans has not been established due to numerous confounders linking MSDP with child outcomes (153). Genetically informed studies designed to disentangle prenatal exposure from familial factors suggest that the independent effect of MSDP on neurodevelopmental risk is either very small (154, 155) or absent (156, 157).

What *is* clear is that *families transmit mental health risk*—through genes, parenting behavior, the rearing environment, and their complex interactions. This means that improving long-term mental health outcomes in children exposed to MSDP requires modification of maternal behaviors other than smoking. The proposed developmental framework which embeds MSDP within the greater landscape of the maternal–child relationship is an important step toward characterizing the insidious processes for which MSDP may simply be a marker. Even a minute possibility for successful preventive intervention justifies scientific investment and collaboration when the risks associated with the *status quo* are so high and so far-reaching.

## Author Contributions

SM drafted the initial manuscript. SM, JD, KW, and LW made substantial contributions to the conception of this manuscript, revising it critically for important intellectual content, and provided final approval for the current version to be published. All authors agreed to be accountable for all aspects of the work in ensuring that questions related to the accuracy or integrity of any part of the work are appropriately investigated and resolved.

## Conflict of Interest Statement

SM, JD, and LW declare that the research was conducted in the absence of any commercial or financial relationships that could be construed as potential conflicts of interest. The Department of Psychiatry at Northwestern University receives contractual fees for KW consultation to Quinn Emanuel Urquhart & Sullivan, LLP (New York City), who represent Pfizer Pharmaceutical Company.
